# Inadequate knowledge about snakebite envenoming symptoms and application of harmful first aid methods in the community in high snakebite incidence areas of Myanmar

**DOI:** 10.1371/journal.pntd.0007171

**Published:** 2019-02-15

**Authors:** Mohammad Afzal Mahmood, Dale Halliday, Robert Cumming, Khin Thida Thwin, Mya Myitzu, Julian White, Sam Alfred, David A. Warrell, David Bacon, Win Naing, Htay Aung, Myat Myat Thein, Nyein Nyein Chit, Sara Serhal, Myat Thet Nwe, Pyae Phyo Aung, Chen Au Peh

**Affiliations:** 1 School of Public Health, University of Adelaide, Adelaide, Australia; 2 School of Public Health, University of Sydney, Sydney, Australia; 3 Ministry of Health and Sport, University of Medicine 1 & Yangon Specialist Hospital, Myanmar; 4 Project Field Team, Myanmar Snakebite Project, Mandalay, Myanmar; 5 Toxinology Department, Women’s & Children Hospital, Adelaide, Australia; 6 Emergency Department, Royal Adelaide Hospital, Adelaide, Australia; 7 Nuffield Department of Clinical Medicine, University of Oxford, Oxford, United Kingdom; 8 Ministry of Health and Sport, Myanmar; 9 Kyaukse District Government Hospital, Kyaukse Township, Myanmar; 10 Regional Department of Public Health, Ministry of Health, Mandalay, Myanmar; 11 Department of Renal Medicine, Royal Adelaide Hospital, Adelaide, Australia; University of Kelaniya, SRI LANKA

## Abstract

**Introduction:**

Every year millions of people in developing countries suffer from snakebite, causing a large number of deaths and long term complications. Prevention and appropriate first aid could reduce the incidence and improve the health outcomes for those who suffer bites. However, many communities where snakebite is a major issue suffer from a lack of information about prevention and first aid measures that a family or community member could take to prevent severe envenoming, complications and poor outcomes. Myanmar suffers from a high burden of snakebites with a large number of deaths. As part of a health services and community development program, a community survey was conducted to identify communities’ knowledge about snakebite and their sequelae, and knowledge and practice about first aid and health services use.

**Method:**

4,276 rural residents of Kyaukse and Madaya townships in the Mandalay region were recruited by cluster sampling, involving random selection of 144 villages and random sampling of 30 households from each village. One adult member of each household was interviewed using a structured questionnaire.

**Results:**

The incidence of snakebite was 116/100,000 people. Respondents reported 15 different types of snakes in the area, with Russell’s Viper, Cobra and Green snakes as the most common. 88% of the people informed that working in the fields and forests was when most of the bites occur. A majority knew about snakebite prevention methods such as wearing long boots. However, only a few people knew about the specific symptoms caused by snakebites. Only 39% knew about the correct methods of first aid. More than 60% mentioned tourniquet as a first aid method, though this may cause significant complications such as ischaemia of the limb. 88% said that they would take a snakebite victim to a government hospital, and 58% mentioned availability of antivenom as the reason for doing this. At the same time, the majority mentioned that traditional methods existed for first aid and treatment and 25% mentioned at least one harmful traditional method as an effective measure that they might use.

**Conclusion:**

The community is aware of snakebites as a major public health issue and know how to prevent them. However, the high incidence of snakebites point to lack of application of preventive methods. The community recognise the need for treatment with antivenom. However, inadequate knowledge about appropriate first aid methods, and a reliance on using tourniquets require a targeted education program. Existing knowledge in communities, albeit insufficient, provides a good starting point for mass media educational campaigns.

## Author summary

Snakebite is a major public health problem, particularly in developing countries in the tropics, and every year millions of people suffer from snakebite causing a large number of deaths and long term complications. Communities’ knowledge about snakebite prevention practices and appropriate basic first aid could reduce the number of snakebites and improve the health outcomes for those who suffer bites. However, many communities where snakebite is a major issue may lack information about prevention and first aid measures that a family or community member could take to prevent severe complications and poor outcomes. Myanmar suffers from a high burden of snakebites with a large number of deaths. We conducted a community survey in two townships to identify communities’ knowledge about snakebite prevention, first aid and health services use. The survey informed that a large majority of people were aware that working in the fields and forests was when most of the bites occur. Similarly, a majority has the knowledge about snakebite prevention methods such as wearing long boots. However, the majority did not know about the correct methods of first aid, with many people mentioning tourniquet as a first aid method. While the community is aware of how to prevent snakebites, the fact that number of snakebites is high points to lack of application of those preventive methods. The inadequate knowledge about appropriate first aid methods with a reliance on using tourniquets informs about the need for public health education programs.

## Introduction

Many people in developing countries suffer from snakebites, causing many deaths and long-term complications. Venomous snakebites cause local and systemic problems such as shock, bleeding, kidney injury, paralysis, infections, long term pituitary dysfunction and local necrosis. Global annual snakebite incidence is estimated as about 4.5 to 5.4 million patients, with more than 100,000 deaths [1]. However, the number of deaths may be substantially higher [2], with 45,000 deaths annually in India alone [3], based on community surveys, which detect higher rates than government hospital statistics.

Prevention and appropriate first aid are important public health measures to reduce incidence and severity of snakebite envenoming. Communities’ knowledge and implementation of snakebite prevention, appropriate first aid, and choice of medical care can help reduce the burden. At present, communities have limited understanding of correct first aid methods. Many choose potentially harmful methods [4]. Inadequate emphasis on public health education, with a resulting failure to promote appropriate preventive and first aid, adds to the challenges of addressing this issue.

Traditional methods for treating snakebite are commonly used [5]. Most of them are useless and some; such as making incisions, sucking the venom, and application of tight tourniquets; are deleterious [6]. In Sri Lanka, despite high levels of formal education and good awareness about snakebite, more than 43% of bite victims sought care from traditional healers [7]. Even those who had relatively easy access to allopathic treatment still sought traditional healing [7]. One reason for the persistent use of techniques that may be useless or downright deleterious, is that some, such as electroshock, cryotherapy, herbs, oils, and suction devices applied to the wound, are still claimed to be effective [8].

One commonly used first aid measures that is to tie a tourniquet around the bitten limb, in the belief that it slows the spread of venom from bite site through veins and lymphatics. In the past, public health educational campaigns promoted the use of tourniquets [8]. The pressure pad technique, originally described by Anker et al [9], utilises a pad tightly applied to the bite site. This technique delayed the leakage of venom into the systemic circulation in Russell’s viper bite victims in Myanmar [10], without the potential for vascular occlusion and limb ischaemia associated with application of a tourniquet. However, tourniquets remain the most commonly used first aid method in developing countries. In Bangladesh, more than 95% of snake bitten patients had applied them [11], in Nepal, 70% of people described the use of tourniquets as appropriate first aid for snakebite [12] and even in Sri Lanka, where communities’ knowledge about snakes and snakebite is relatively sound, 35% of snakebite victims applied tourniquets [13].

Snakebite is a major public health issue in Myanmar with more than 15,000 bites and more than 1,000 deaths annually [14]. We conducted a community survey in the Mandalay region of Myanmar to identify population-based incidence and communities’ knowledge of snakebite, prevention, first aid and use of health services. The survey was conducted as part of a health system development project of the Myanmar Ministry of Health and Sports and Ministry of Industry together with the University of Adelaide and other Australian and international institutions funded through an Australian Government foreign aid grant. Information about community knowledge and practices is essential for an effective health education program and an effective health system response.

## Method

The survey was conducted in the rural townships of Kyaukse and Madaya in the Mandalay region, which is among the highest snakebite incidence regions in Myanmar. It was conducted in rural areas as most snakebites occur in rural farming communities. Relatively few people live in the urban town centres of these townships (Kyaukse 16%, Madaya 9%). Most live within the smaller agricultural villages that surround them. Population of both Madaya and Kyaukse is about 258,000 each. A majority of the 15–64 years of age people, 51.4% in Kyauske and 54% in Madaya work in agriculture, forestry and fishing [15]. Main crops include rice, wheat, legumes and vegetables. The sampling unit was the household, in which an adult member was interviewed in each of those selected. The sample size was 4,500 households with about 20,000 household members.

Cluster sampling was used, as recommended in the WHO Vaccination Coverage Cluster Surveys [16], with three stages of sampling i.e. (i) stratification by township, (ii) random selection of 75 villages from each of the two townships, (iii) random sampling of 30 households from each village selected from lists of households provided by the local government health departments. Census data on the population of each village were used for stage ii so that random sampling of villages was done with a probability proportional to their sizes.

An interviewer-administered questionnaire was used to collect data. The structured questionnaire targeted the respondents’ knowledge of local snake fauna, the symptoms of snakebite, preventive and first aid methods, traditional healing and of health service access. Some of the questions were open-ended so as to capture the potential variety of practices and knowledge of the community members (Table 1).

**Table 1:** Examples of survey questions about knowledge and practice.

Question	Responses
Can you tell me names of the different snakes that live here in this area?	Russell’s viper, Cobra, Krait, Green Snake, Others (specific), Don’t know
Which activity is most associated with people getting bitten in this area?	Lying down, resting or sleeping, while in the toilet, walking home from the fields, fishing, cutting wood, tending animals, farming, Others (specify)
What are the symptoms of someone who has been bitten by a ***Russell’s Viper***?	*Write the answers in respondents words*
What is the treatment for a patient who has been bitten by a snake and is developing symptoms?	*Write the answers in respondent’s words*
If someone from this village were bitten by a snake today, where would you first take them to get treatment, including first aid?	Sub Centre, Rural health centre, station hospital, township hospital, district hospital, private clinic, Traditional Healer, Monastery/Pagoda, Mandalay General Hospital, Private hospital, Charity clinic–Brahamaso [a humanitarian aid organisation], Would not take them anywhere, Other
Which methods do you use regularly to prevent getting bitten by a snake? (*Do not prompt*. *Multiple answers possible)*	Wear long boots, Wear gloves, Use a torch or light when it is dark First use a stick to check before working in the area, Put mosquito nets around beds, Do not sleep on the ground, Do not put hands/feet in holes/dark places where you cannot see, Keep dogs to warn when snakes are nearby, Clean bushes, Use machines when farming, Does not use any, Other (specify

The statistical analysis was conducted using SPSS (IBM SPSS Statistics Version 24) after the survey data were weighted for respondent sampling probability. All numeric findings are adjusted for the three stage sampling methodology. Chi-squared test was used to calculate p values to assess difference between the knowledge among men and women, between respondents who lived in high snakebite incidence villages and those who lived in low incidence villages, and the respondents with and without family history of snakebite during the last ten years.

The research was conducted with ethics approval from the Human Research Ethics Committee at the University of Adelaide and Ethics Committee at the Department of Medical Research at the Ministry of Health in Myanmar.

## Results

The survey was conducted in 74 villages in Kyaukse township and 70 villages in Madaya townships, with 4,276 respondents. The participation rate was 94%. Half (49,9%) of respondents were women. The majority, 41.9%, of the respondents were 41–60 years of age, 35.5% were 18–40 years and 20.5% were above 60 years of age.

Mandalay region is a high snakebite incidence area. The respondents reported that among these 4,276 households 24 people were bitten in the last one year. Incidence, types of snakes and activities associated with the snakebite have been reported in a previous publication [14].

### Knowledge about symptoms

Very few respondents had an adequate understanding of the symptoms caused by Russell’s Viper bite, the snake responsible for most envenomings in this region. Similarly, only a few respondents had accurate knowledge about the symptoms caused by cobra bite. Bleeding, which is among the main symptoms of Russell’s Viper envenoming, was mentioned by only 2.9% of respondents, while neurological symptoms, which rarely if ever occur with Russell’s Viper bite, were mentioned by about 30% of respondents. The majority of the respondents appeared not to have adequate knowledge about the specific symptoms of snakebite. However, more than half mentioned, in generic terms, that snakebite causes serious health consequences (Tables 2 & 3). There was no difference between survey respondents who reported that one of their family members suffered from snakebite during the last ten years, and those with no direct exposure to the consequences of snakebite.

**Table 2:** Community Members’ knowledge about Russell’s Viper symptoms.

Symptoms	RV bite All Respondents % (n:4276)	RV bite—respondents who reported a snakebite in their family % (n:252)
Serious Health Problem (non specific)	50.3	30.6
Local Symptoms at bite wound	25.0	17.3
Neurological	29.6	18.4
Bleeding	2.9	0.5
Renal	2.6	0.8
Lethargy	10.7	5.1
Nausea	9.3	4.7
No Answer	12.9	12.4

**Table 3:** Community Members’ knowledge about Cobra bite symptoms.

Symptoms	All Respondents % (n:427)	Respondents who reported a snakebite in their family (n—252)
Serious Health Problem (non specific)	44.0	31.3
Local Symptoms at bite wound	10.6	7.4
Neurological	10.6	10.8
Bleeding	0.4	0
Renal	10.2	0
Lethargy	3.3	2.2
Nausea	2.6	2.5
No Answer	38.3	36.8

There was no statistically significant difference between men and women’s knowledge of symptoms. Similarly, there was no statistically significant difference in the knowledge of those who were less than 40 years of age and those above 40 years. The respondents from 40 villages reported three or more snakebites for each of those 40 villages during the last ten years. There was no statistically significant difference in knowledge of respondents from those villages which reported 3 or more snakebites and those villages which had none.

### Knowledge about prevention

Many respondents had knowledge about some of the preventive methods, such as wearing long boots (79.4%), using a torch when in dark (58.9%), using a stick to check before working [for example, collecting harvested] crops (28.9%), and clearing bushes around homes (11.5%). A few of the respondents mentioned wearing gloves, using mosquito bed nets, and not sleeping on the floor. A few others mentioned saying prayers or offering goods to spirits to help prevent snakebites. There was no statistically significant difference in prevention methods knowledge of respondents from those villages which reported 3 or more snakebites and those villages which had none.

### Knowledge about first aid

When asked which first aid method they would provide to a snakebite victim, many respondents (62%) mentioned application of a tourniquet (Table 4). Best practice first aid methods include pad and bandage over the bite site, splinting the limb, carrying the patient and not letting them walk if the bite is on lower limb. Most (72%) respondents did not mention any of these best practice methods.

**Table 4:** Community members’ knowledge about the first aid.

First Aid Method	Respondents % (n:4276)
Tourniquet	61.8
Cut the wound	2.0
No answer	10.9
Apply herbal products	2.1
Feed herbal products	2.0
Pad and Bandage	13.3
Splint the limb	12.4
Carry the victim & don’t allow walk	12.0
Put arm in a sling	0.9
Support breathing	0.4
Get help from health staffs/neighbours	1.6

### Traditional method in use

When asked if there were any effective traditional methods for treating snakebite, 37.4% answered that there were some effective methods to treat snakebite. One in four mentioned one or more harmful traditional method such as cutting the bite site. Kyaukse township has a larger district hospital, and relatively fewer people from this region mentioned potentially harmful traditional methods compared to those from Madaya township. In Madaya 42.9% (CI 38.0–47.8) people mentioned traditional methods compared to 31.1% (CI 27.4–35.1) in Kyaukse. Harmful methods were mentioned by 29.0% and 20.0% people in Madaya and Kyaukse respectively. Harmful traditional methods included sucking out the blood from the bite site, cutting or tattooing around the bite, burning the bite area, drinking something to cause vomiting, and rubbing/massaging traditional medicine into the bite area. Non harmful traditional methods included using a black stone (considering that the stone is applied without incising the wound), drinking holy water, saying prayers, drinking coconut water, or applying a dead chick to the bite site.

### Health services use & knowledge about treatment

When asked where they would first take the snakebite victim, a large majority (88%) of the respondents mentioned the teaching hospital in nearby Mandalay city, or a township hospital or smaller station hospital near their village. Another 4% mentioned government health centres. Only 1.6% said that they would take the snakebite victim first to a traditional healer. The main reasons for accessing government health facilities included availability of antivenom (58.3%), and the better quality of care (19.7%). Only 10.8% of the respondents answered that their reason for using the health centre/hospital was the short distance and travel time to the health centre/hospital in their areas. While 55.2% mentioned one or more best practice treatment methods, many mentioned applying tourniquet (44%) and one or more other harmful methods such as cutting or incising the bite site (13%).

## Discussion

### Good knowledge about preventive methods but inadequate application

The high incidence of snakebites despite people’s having adequate knowledge about prevention suggests that preventive methods are not being practised. Despite a high number of bites at the population level, snakebite is a rare event for an individual. Therefore, people may not perceive themselves at risk of being bitten. Preventive action requires that people not only perceive a threat of high severity but also perceive themselves at risk of suffering from that health problem [17]. Health education campaigns could use the foundation of good preventive knowledge to help people perceive the risk and turn knowledge into practice. Other reasons such as difficulty wearing boots in a hot tropical climate or wearing them while working in rice fields have been noted, but light weight, low cost boots proved to be acceptable to farmers in a pilot study [18].

### Poor knowledge about symptoms

Communities’ knowledge about snakebite symptoms is very limited. This reflects the fact that, despite snakebite being a major public health issue, health education has been limited. Even those with a snakebite in the family have limited understanding of symptoms, including renal problems, despite the fact that the majority of bites in this area are by Russell’s Viper whose bites carry a high risk of causing acute kidney injury. This lack of awareness about renal and other specific effects may also reflect the general level of health literacy in the community. While community’s lack of knowledge about symptoms may not be so problematic as far as treatment at health facilities is concerned where healthcare provider would be able to differentiate, in most of the cases, between cobra and Russell’s viper bite, it may cause delays in accessing care; for example, as many believed that Russell’s Viper bite causes neurological symptoms, they may not seek care if these symptoms system do not appear. Community-based health education and mass media-based awareness campaigns need to focus on delivering messages about symptoms and their implications for the need for seeking appropriate care.

### Incorrect first aid methods with potentially poor outcomes

Many mentioned tourniquets as a first aid measure. Observations at the hospitals in the region confirm that many victims arrive at the hospitals with a tourniquet applied. Tourniquets and ligatures have long been used by indigenous peoples to decrease the spread of venom [19]. Avau considers that the reasons for the lack of use of the pressure pad technique is that it has only recently been introduced to health education and that some literature still doubts its effectiveness causing uncertainty among health educators and communities [8]. There is a need for mass media-based awareness campaigns to alert people to the damaging effects of a tightly applied tourniquet.

### Traditional healing methods use and their potential implications

Although few respondents answered that they would take a victim to a traditional healer, many reported knowing about traditional healing methods that were potentially harmful. Relatively fewer people from the township that had a larger hospital with a reputation for effective management of snakebite reported use of traditional healing methods. This probably reflects the impact of community trust in the capacity of formal health care services in that district. Access to services at a well-respected larger hospital with effective care and with abundant supplies of antivenom, potentially affects people’s use of traditional services. However, a previous qualitative participatory research project conducted by us in three of the villages where our current survey was conducted, suggested that traditional healers were trusted and used despite improved access to modern biomedical treatment and availability of antivenom [5]. Traditions are important to people as they are rooted in faith and cultural belief systems, local rituals, and practices promoted by loved ones and elders. However, it is important that the communities are aware that these practices are potentially harmful and that visiting a traditional healer may cause delays in reaching a health facility where antivenom is available if needed. Therefore, it is important that a sensitive but assertive approach is taken whereby allopathic modern medicine is not seen to be in competition with traditional methods, but the community is informed of the need for careful assessment of the risks associated with some of these practices. Empowering people with simple scientific knowledge about the cause of various symptoms will help more people to utilise health facilities early and avoid using harmful traditional methods.

### Limitation

This survey was conducted in townships close to a large city, with health care and antivenom available at many large government hospitals. This would not affect findings about knowledge about prevention and first aid, but might have affected responses to questions such as ‘where would you take a snakebite victim’. The interviews were conducted by primary care staff who might have caused response bias for the questions about use of traditional healers.

Despite the above-mentioned limitation, the survey, based on interviews with a large number of community women and men, highlighted the gaps in communities’ knowledge about prevention, first aid and treatment. The fact that despite the high prevalence of snakes and snakebites in the research area, the community members had inadequate knowledge, indicates that existing health promotion and health education initiatives have had little impact on understanding and practices. There is an immediate need for health education campaigns about snakebite prevention and first aid methods that target primary care workers in the public sector and communities through those primary care workers. While effectiveness of mass media educational campaigns for communities’ knowledge about snakebite prevention and first aid is not assessed yet, considering the need to reach out to large populations in high snakebite incidence regions of Myanmar FM radio and television campaigns might also be considered, and their impact evaluated, for improved knowledge about prevention and first aid. Health education programs must also involve local community women and men and community based organisations in planning and implementing such programs, as it helps avoid cultural bias; a challenge that besets many modern health program [20]. In this research setting, participatory assessment and planning involving locals informed that such participation leads to an in depth understanding about the local beliefs and practices [5].

## Supporting information

S1 STROBE Checklist Checklist of items for this observational study. (DOCX)
