# Supplementary material for: Inadequate knowledge about snakebite envenoming symptoms and application of harmful first aid methods in the community in high snakebite incidence areas of Myanmar
Source: PLoS Negl Trop Dis. 2019 Feb 15;13(2):e0007171. doi: 10.1371/journal.pntd.0007171 (PMC6395000; doi:10.1371/journal.pntd.0007171)
Supplement: S1 STROBE Checklist — (DOCX) [file pntd.0007171.s001.docx]

STROBE Statement for ***cross-sectional studies***

Community Knowledge about Snakebite Prevention and First Aid in Myanmar

|  | Item No | Recommendation |
| --- | --- | --- |
| **Title and abstract** | 1 | (*a*) Line 2 |
|  |  |  |
| Introduction | | |
| Background/rationale | 2 | Lines 82-121 |
| Objectives | 3 | Lines 116-117 |
| Methods | | |
| Study design | 4 | Lines 131-135 |
| Setting | 5 | Lines 124-129 |
| Participants | 6 | Lines 127-129, 154-156 |
| Variables | 7 | Lines 137-144 |
| Data sources/ measurement | 8* | Lines 137, 146-148 |
| Bias | 9 | Lines 277-281 |
| Study size | 10 | Lines 127-129 |
| Quantitative variables | 11 | Lines 137-144 |
| Statistical methods | 12 | Lines 146-148 |
|  |  | Analytical method for sampling strategy – line 147 |
|  |  | Sub-groups and interactions – Not Applicable |
|  |  | Sensitivity Analysis – Not Applicable |
|  |  |  |
| Results | | |
| Participants | 13* | Lines 155-156 |
| Descriptive data | 14* | Lines 161-226 |
| Outcome data | 15* | NA |
| Main results | 16 | Lines 153-226 |
| Other analyses | 17 | NA |
| Discussion | | |
| Key results | 18 | Lines 229-274 |
| Limitations | 19 | Lines 277-281 |
| Interpretation | 20 | Lines 243-246, 253-255, 261-266 |
| Generalisability | 21 | Lines 283-2289 |
| Other information | | |
| Funding | 22 | \| DFAT, Australia. Information provided in online submission system \| \| --- \| |

*Give information separately for exposed and unexposed groups.

**Note:** An Explanation and Elaboration article discusses each checklist item and gives methodological background and published examples of transparent reporting. The STROBE checklist is best used in conjunction with this article (freely available on the Web sites of PLoS Medicine at http://www.plosmedicine.org/, Annals of Internal Medicine at http://www.annals.org/, and Epidemiology at http://www.epidem.com/). Information on the STROBE Initiative is available at www.strobe-statement.org.
